# Turning natural adaptations to oncogenic factors into an ally in the war against cancer

**DOI:** 10.1111/eva.12608

**Published:** 2018-03-14

**Authors:** Marion Vittecoq, Mathieu Giraudeau, Tuul Sepp, David J. Marcogliese, Marcel Klaassen, François Renaud, Beata Ujvari, Frédéric Thomas

**Affiliations:** ^1^ Institut de Recherche de la Tour du Valat Arles France; ^2^ CREEC/MIVEGEC IRD CNRS University of Montpellier Montpellier France; ^3^ School of Life Sciences Arizona State University Tempe AZ USA; ^4^ Centre for Ecology & Conservation College of Life and Environmental Sciences University of Exeter Penryn UK; ^5^ Department of Zoology University of Tartu Tartu Estonia; ^6^ Aquatic Contaminants Research Division Water Science and Technology Directorate Environment and Climate Change Canada St. Lawrence Centre Montreal QC Canada; ^7^ Fisheries and Oceans Canada St. Andrews Biological Station St. Andrews NB Canada; ^8^ School of Life and Environmental Sciences Centre for Integrative Ecology Deakin University Deakin Vic. Australia; ^9^ School of Biological Sciences University of Tasmania Hobart TAS Australia

**Keywords:** cancer resistance, ecotoxicology, experimental evolution, pollution, wildlife

## Abstract

Both field and experimental evolution studies have demonstrated that organisms naturally or artificially exposed to environmental oncogenic factors can, sometimes rapidly, evolve specific adaptations to cope with pollutants and their adverse effects on fitness. Although numerous pollutants are mutagenic and carcinogenic, little attention has been given to exploring the extent to which adaptations displayed by organisms living in oncogenic environments could inspire novel cancer treatments, through mimicking the processes allowing these organisms to prevent or limit malignant progression. Building on a substantial knowledge base from the literature, we here present and discuss this progressive and promising research direction, advocating closer collaboration between the fields of medicine, ecology, and evolution in the war against cancer.

## 1 INTRODUCTION

Although medical and evolutionary sciences have traditionally developed in relative isolation (Williams & Nesse, 1991), applying evolutionary principles and approaches to understand the emergence and development of cancer has gained significant international recognition over the last decade (Aktipis & Nesse, 2013; Greaves, 2007; Thomas et al., 2013; Ujvari, Roche, & Thomas, 2017). A flagship direction of this research area is the identification of natural cancer suppressive mechanisms, knowing that evolution had eons to fine‐tune adaptations to the fitness cost of malignant selfish cells. Cancer is indeed a disease that developed along the transition from unicellular to metazoan life, approximately one billion years ago (Aktipis & Nesse, 2013; Merlo, Pepper, Reid, & Maley, 2006; Nunney, 2013). Because multicellular individuals with unregulated cell division were at a selective disadvantage over those that were able to prevent uncontrolled cell proliferation, strong constraints on somatic evolution to suppress cancer have evolved along with multicellularity (Aktipis & Nesse, 2013).

In this context, the absence of a positive relationship between size/life expectancy and cancer incidence across species, known as Peto's paradox, has attracted the attention of many evolutionary biologists (Caulin & Maley, 2011). Indeed, if every cell has some chance of becoming cancerous, large long‐lived organisms should have an increased risk of developing cancer compared to small, short‐lived organisms. The lack of correlation therewith suggests that the mechanisms of cancer resistance must have been more strongly selected in large and long‐lived species (Caulin & Maley, 2011; Nunney, Maley, Breen, Hochberg, & Schiffman, 2015; Roche et al., 2012). Accordingly, it has for instance been shown that large vertebrates such as elephants have 20 copies of TP53 (humans have only one), horses seem to have larger number of T‐cell differentiation protein (MAL) genes, and bats (that live unexpectedly long given their small body size) have amplified F‐box protein 31 (FBXO31) (Caulin, Graham, Wang, & Maley, 2015; Harris, Schiffman, & Boddy, 2017; Kokko, Schindler, & Sprouffske, 2017).

Recently, Ducasse et al. (2015) argued that apart from body size and longevity, additional ecological, environmental, and behavioral factors should also be considered when assessing cancer prevalence, and attempting to identify species with resistance to cancer. Major steps have been made toward this goal through the study of mammal species that seem to be free from cancer or at least exhibit extremely low prevalence of tumor occurrence. This has recently been synthesized by Tollis, Schiffman, and Boddy (2017) who discussed the mechanisms of cancer resistance that have so far been discovered in two mole rat species, *Heterocephalus glaber* (see, e.g., Seluanov et al., 2009; Tian et al., 2013) and *Spalax* sp. (e.g., Manov et al., 2013; Schmidt, Hangmann, Shams, Avivi, & Hankeln, 2016). The same approach has led to important insights into the mechanisms that confer partial cancer resistance in humans suffering from different forms of growth hormone receptor deficiency (Guevara‐Aguirre et al., 2011; Shevah & Laron, 2007). However, despite recent progress toward greater convergence and dialogue between scientists working on oncology, ecology and evolutionary sciences much remain to be done to achieve full integration of these disciplines.

Here, we propose that a promising research direction, still largely underexplored at the moment, is the search for cancer suppressive mechanisms that may have evolved in organisms living in environments that favor cancer emergence and progression. Similar to a lack of correlation between life expectancy and cancer incidence that led to Peto's paradox, a lack of correlation between cancer incidence and rate of exposure to pollutants, especially mutagens and carcinogens (hereafter called environmental oncogenic factors or EOF), might also hold true. Such lack of correlation, if present, might suggest that evolution has produced solutions to avoid and/or control malignant problems in EOF‐exposed populations. Below we provide information suggesting that both field and experimental evolution studies may be promising avenues to the discovery of novel mechanisms of cancer resistance that could potentially enable novel cancer therapies.

## 2 AVAILABLE EVOLUTIONARY‐ECOTOXICOLOGY KNOWLEDGE

Life on earth has evolved under the ubiquitous presence of EOF including chemicals present in air, water, and sediment such as polycyclic aromatic hydrocarbons. Various types of radiation have also played a significant role as EOF, challenging life processes (Aarkrog, 1990; Beresford & Copplestone, 2011; Mothersill, Rusin, & Seymour, 2017; Sivani & Sudarsanam, 2012). Moreover human activities have resulted in major, large‐scale environmental modifications throughout our history, with the scale and speed of anthropogenic impacts exponentially increasing over the past century (Lebarbenchon, Brown, Poulin, Gauthier‐Clerc, & Thomas, 2008; Vitousek, Mooney, Lubchenco, & Melillo, 2008).

The impact of ecosystem contamination by EOF has been studied since the 1970s due to growing concern about the consequences of increasing environmental pollution on ecosystem function and species extinction (Butler, 1978; Truhaut, 1977). At the end of the 1990s, this field transitioned when it became obvious that the impact of EOF could not just be evaluated at the scale of an individual or a generation but should also be assessed in terms of consequences on population evolution: Evolutionary ecotoxicology was born (Bickham & Smolen, 1994; Depledge, 1998). Since then, the evolutionary consequences of EOF ecosystem contamination have been explored at different levels (i.e., genetic, epigenetic, and developmental) forming an abundant literature, albeit focusing mainly on aquatic species (e.g., Oziolor, Bigorgne, Aguilar, Usenko, & Matson, 2014; Reid et al., 2016; Wirgin et al., 2011). The development of these studies led to the discovery of a large diversity of adaptation mechanisms allowing populations to survive and reproduce in highly contaminated sites. Here, we suggest that this considerable amount of data and knowledge could be used and developed with a focus on cancer resistance and tolerance mechanisms. This research axis seems to have attracted little interest so far, notably due to the complexity and diversity of pathways involved that could be easier to unravel with rapidly improving analytical techniques (see, e.g., Nesnow, 2013).

**Figure 1:**
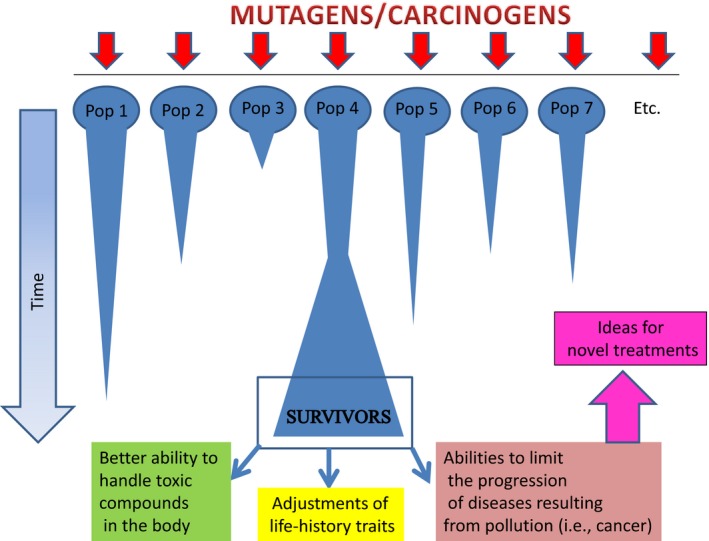
Detecting anticancer adaptations from experimental selection

This topic is still in its infancy. We still need to determine why, when exposed to mutagenic substances, certain species display higher mutation rates and/or show more rapid adaptive responses (e.g., DeWoody, 1999; Eeva, Belskii, & Kuranov, 2006; Rotchell, Lee, Chipman, & Ostrander, 2001). For example, large amounts of standing genetic diversity may be an important factor facilitating rapid adaptations, as observed in the Atlantic killifish (Reid et al., 2016, 2017). Distinguishing between physiological acclimation and evolutionary heritable changes is also crucial (e.g., Fisker, Sørensen, Damgaard, Pedersen, & Holmstrup, 2011; Hamilton, Rolshausen, Webster, & Tyler, 2017; Mousseau & Møller, 2014). Moreover, we need to determine the extent to which species, when naturally or artificially exposed to EOF, are selected (i) to better handle toxic compounds in the body, (ii) to limit their fitness impact through an adjustment of life‐history traits, and/or (iii) to select mechanisms that limit the occurrence and progression of EOF‐induced diseases, like cancer. To make the distinction between the different adaptive strategies species follow when exposed to EOF is potentially important from a medical perspective. Obviously, it is the latter third option that bears the promises of yielding novel treatments against cancer (Figure 1). Both field and experimental studies, or a combination of the two, could be used in this context.

## 3 ADVANTAGES AND DRAWBACKS OF FIELD AND EXPERIMENTAL STUDIES

Selection of genetically inherited tolerance in populations exposed to EOF contamination, either of natural or anthropogenic origin, has been highlighted in a large range of aquatic and a few terrestrial species throughout the world (Giulio & Clark, 2015; Johnson & Munshi‐South, 2017; Medina, Correa, & Barata, 2007; Oziolor & Matson, 2015; Whitehead, Pilcher, Champlin, & Nacci, 2012; Wirgin & Waldman, 2004). Field studies have highlighted the existence of such adaptations arising at time scales varying from millennia to a few years only.

For instance, *Drosophila melanogaster* from high altitude display local adaptation to cope with DNA‐damaging ultraviolet, via solutions involving polymorphisms in DNA‐repair genes (Svetec, Cridland, Zhao, & Begun, 2016). Selection for ultraviolet tolerance is also observed in some fungi and bacteria that are exposed to UV radiation for part of their life cycles (Paul & Gwynn‐Jones, 2003). In both *Daphnia* and humans, pigmentation is considered as an adaptation to UV radiation (Jablonski & Chaplin, 2010; Tollrian & Heibl, 2004). In those cases, selection pressure has acted over thousands of generations as is typically the case for adaptations related to EOF of natural origin.

By contrast, evolution processes associated with anthropogenic pollution can occur on a temporal scale of years and within few generations only (e.g., Bélanger‐Deschênes, Couture, Campbell, & Bernatchez, 2013; Klerks & Levinton, 1989; Knapen, Bervoets, Verheyen, & Blust, 2004; Theodorakis, Blaylock, & Shugart, 1997). For instance, in the benthic oligochaete *Limnodrilus hoffmeisteri* inhabiting a metal‐polluted site in Foundry Cove (New York), resistance to metal pollution evolved within 30 years only (Klerks & Levinton, 1989). However, although currently rapidly accelerating, anthropogenic impacts on the environment, such as mining related contamination, have been ongoing for centuries. As an illustration, in England, “metal‐river” trout populations are genetically distinct from “clean‐river” populations despite being geographically in close proximity. A split dating back to Medieval times when local mining activity was highly intensive (Paris, King, & Stevens, 2015).

Field studies provide the opportunity to examine the evolution of naturally occurring EOF tolerance mechanisms in a very large diversity of cases. The heritable tolerance mechanisms involved can either arise from the initial genetic pool of the focal population, the mutation induced by EOF, the genetic pool of neighboring populations, or a combination of these. This represents both limits and advantages. Indeed, in a closed population facing a new contamination, genetic diversity is expected to decrease due to the mortality or low fecundity of nonresistant individuals. Such a decrease has been observed in a number of cases. For example, in Canada, wild yellow perch (*Perca flavescens*) living in lakes impacted by mining activities experienced a significant reduction in population genetic diversity following pollution (Bourret, Couture, Campbell, & Bernatchez, 2008). Nevertheless, in most cases, genetic diversity either increases or remains stable following contamination of ecosystems. In these cases, increased mutation and/or immigration rates compensate or exceed the loss of diversity due to contamination impacts on the initial population. As an illustration, no impact on genetic diversity was observed in American mink (*Neovison vison*) populations facing polychlorinated biphenyl contamination in Belgium (Wirgin, Maceda, Waldman, & Mayack, 2015). The same was true for bank voles (*Clethrionomys glareolus*) living in Chernobyl in irradiated areas (Baker et al., 2001). Moreover “sink‐like” populations have been identified in different studies following EOF exposure. For example, in North Carolina, the genetic diversity of redbreast sunfish (*Lepomis auritus*) was higher in rivers impacted by pulp mill effluent discharge (Theodorakis, Lee, Adams, & Law, 2006). Similarly, Giska, Babik, van Gestel, van Straalen, and Laskowski (2015) showed that their most polluted study site in Poland had the population of rove beetles with the highest genetic diversity. Yet, it is important to note that in such sink‐like populations, only few individuals may contribute to the next generation, which means that if the population becomes isolated its genetic diversity could decrease rapidly.

The observed high mutation and immigration rates may dilute local adaptations and thus hamper the identification of cancer resistance mechanisms. On the other hand, these high mutation and immigration rates may also allow for the selection of relevant adaptations from a larger than the initial genetic pool. Once established, these specific adaptations may incur a fitness cost in the original unpolluted environment. Various populations of pollutant‐tolerant killifish (*Fundulus heteroclitus*) experienced such costs in clean water, including higher mortality associated with infectious diseases and higher rates of acute hypoxia, when compared to pollutant susceptible congeners (reviewed in Whitehead, Clark, Reid, Hahn, & Nacci, 2017). Such costs may limit the natural diversity of selected EOF resistance mechanisms. But they are also interesting in themselves as they identify the possible drawbacks of the EOF resistance mechanisms. However, in some cases, these costs could be due to hitchhiked genetic regions, which are often associated with rapid selective sweeps (Schiffels, Lässig, & Mustonen, 2014; Shiina et al., 2006). These hitchhiked genetic regions could potentially be removed experimentally to test for the selective value of the relevant adaptations in isolation.

Experimental evolution is a complementary approach that consists in the use of laboratory or controlled field manipulations to investigate evolutionary processes. It has helped in proving that EOF resistance in multicellular organisms is often heritable and can rapidly evolve. For instance, selection experiments on the Foundry Cove worms (*L. hoffmeisteri*) indicated that 1–4 generations are enough for resistance to metal pollution to evolve (Klerks & Levinton, 1989). Similarly, an artificial selection experiment for cadmium resistance in the least killifish, *Heterandria formosa*, showed that after only six generations of selection, fish survived about three times as long as control‐line fish when exposed to cadmium (Xie & Klerks, 2003). The evolution of cadmium tolerance has also been demonstrated by laboratory experiments on daphnid populations (Ward & Robinson, 2005). Indeed, the costs of coping with interspecific interactions (e.g., predation, competition, parasitism) that are removed in experimental settings may, because of trade‐offs existing under natural conditions, prevent the evolution of adaptations to toxicants (Beketov & Liess, 2006; Foit, Kaske, & Liess, 2012). In artificial selection experiments, laboratory conditions provide an environment less affected by these constraints, thereby increasing the probability of specific adaptations to evolve and to be detected. Experimental approaches give the opportunity to intensify the selection of already known suppressive mechanisms (e.g., additional copies of TP53 as observed in elephants), but could also potentially lead to the emergence of novel ones (i.e., existing in natural populations but not yet discovered, or, indeed, fully novel ones evolving during the experiment but not yet existing in the wild). The latter scenario could be expected if the artificial selection exerts stronger coefficients of selection than any natural or anthropogenic processes in the field.

Furthermore, combining field and experimental approaches is an established and promising avenue to identify EOF adaptations in wild populations. Individuals from polluted and clean sites can be collected and then studied under controlled conditions. In this way, the variation of the tolerance/resistance capacities can be studied over large contaminant‐dose gradients and the effect of acute versus chronic exposure compared. In midges (*Chironomus riparius*), individuals were collected in the field in polluted and clean sites and then reared for six generations in the laboratory. Chronic responses of the studied populations did not consistently converge with acute responses to cadmium exposure (Pedrosa et al., 2017). Studying adaptation mechanisms over several generations is also important to assess their stability and the stability of their function over time. In the same midge population, clutch size and female body weight changed across generations in response to the highly toxic biocide tributyltin (Vogt et al., 2007).

Insight may be gained by examining the combined effects of contaminants and parasites/disease on animals. Such effects may be additive, negative, or neutral depending on the host and contaminant (Marcogliese & Pietrock, 2011), and may be considered analogous to the effects of environmental stressors and cancer on organisms. How organisms physiologically respond to natural and anthropogenic stressors may also be illuminating. For example, it was observed that in yellow perch, two larval trematodes induced oxidative stress, but only at polluted sites. Yet, infection levels were never higher at those sites than at reference sites (Marcogliese, Dautremepuits, Gendron, & Fournier, 2010). Thus, it has been suggested that contaminants may affect tolerance, but not resistance, in this particular system, possibly providing a framework for further examination of immunosuppression elsewhere (Marcogliese & Pietrock, 2011; Marcogliese et al., 2010).

There have been few studies of multigenerational effects of parasites and contaminants on organisms. However, *Daphnia* once again has proven to be a useful model system. Combined effects of the pesticide carbaryl and the bacterial parasite *Pasteuria ramosa* on survival and population growth rate on *Daphnia magna* were synergistic on survival and population growth rate (Coors & De Meester, 2008). Exposure to the same pesticide enhanced the virulence of *P. ramosa* and the microsporidium *Flabelliforma magnivora* (Coors, Decaestecker, Jansen, & De Meester, 2008). However, fitness of *P. ramosa,* measured as reproductive output, actually decreased when daphnids also were exposed to carbaryl (Coors & Meester, 2011).

## 4 AVAILABLE METHODS AND PROMISING RESEARCH AVENUES

So far, field and experimental evolutionary‐ecotoxicology approaches have focused on the understanding of mutation, selection, and spatial structure in EOF‐exposed populations. Yet, the evolution of cancer suppressive mechanisms has rarely been their focus (but see Sprouffske, Merlo, Gerrish, Maley, & Sniegowski, 2012). Possibly this is due to the complexity of the factors involved in the resistance and tolerance to carcinogens. Yet, this is unfortunate, as long‐term population exposure to mutagenic and carcinogenic substances may be expected to efficiently select individuals whose fitness is, by one way or another, less affected, or not affected at all, by cancer burden. Moreover, the rapidly evolving analytical methods, including full‐genome population resequencing and comparative transcriptomics (e.g., Oziolor, Bickham, & Matson, 2017; Reid et al., 2016, 2017) that allow the unraveling of complex “toxic pathways”, linking EOF exposure to its consequences, and that can deal with large datasets, may readily enable such studies.

When candidate genes are already identified, population genetics can help in revealing their association with adaptations to EOF exposure. In the flounder *Platichthys flesus*, a study on the polymorphism of the known tumor suppressor gene p53 across populations living in highly EOF‐contaminated versus reference estuaries showed a significantly higher diversity in polluted sites (Marchand et al., 2010). More powerful techniques such as restriction site‐associated DNA sequencing (RADseq) allow screening the genome of individuals from EOF‐adapted and control populations to highlight polygenic selection. Using this approach, Laporte et al. (2016) identified a total of 142 and 141 covarying markers discriminating European and American eels (*Anguilla anguilla and Anguilla rostrata)* from “control” versus “polluted” sampling localities. Full‐genome population resequencing is yet another promising method, which is rapidly becoming more affordable and which can be used to detect anticancer adaptations. As an example, the analysis of 384 whole killifish genome sequences and comparative transcriptomics allowed the identification of the aryl hydrocarbon receptor (AHR)‐based signaling pathway as a shared target of selection, contributing to the adaptation of individuals to normally lethal levels of pollution in urban estuaries (Reid et al., 2016). Yet, to inspire future cancer treatment development, the identification of genes that are associated with adaptation to EOF should be coupled with thorough analyses enabling the understanding of the complex pathways involved. As an illustration of this, it was through the elaborate coupling of genetic, physiological, chemical, and histological analyses that it was shown that killifish living in an area contaminated with polycyclic aromatic hydrocarbons (PAHs) were resistant to the carcinogenic effects of PAHs (Wills et al., 2010). The AHR‐based signaling pathway seemed to be involved in this pollution resistance, as carcinogenesis requires metabolic activation by enzymes, including enzymes from the cytochrome P450 (CYP)1 family, this activation being mediated by the AHR to which PAHs binds. From these findings, the conclusion could be drawn that if the gene coding for AHR synthesis is inactivated, as proven in mice, individuals are protected against PAH‐induced carcinogenesis (Shimizu et al., 2000). However, another study in mice suggested that, in the absence of a xenobiotic ligand, the AHR gene can function as a tumor suppressor gene (Fan et al., 2010). The above highlights the complexity of the studies we advocate, but that with the inclusion of transcriptomics, proteomics, and metabolomics, we will ultimately be able to better understand phenotypic linkages to genotypes (Oziolor et al., 2017). Understanding that may prove crucial to enable the integration of ecotoxicological knowledge in human cancer research.

So far we have not addressed variations in levels and duration of EOF exposure, whereas these may impact the selective pressure associated with EOF exposure and accordingly affect the evolution of tumor resistance mechanisms. In this respect, hormesis could be an important phenomenon to study, as it could shape the link between EOF exposure and tumor resistance evolution. Hormesis describes the biphasic dose‐dependent response to toxic substances or other pollutants (i.e., radiation) that have stimulatory or beneficial effects at low doses, but detrimental effects at high concentrations (Calabrese & Baldwin, 2003; Southam & Erlich, 1943). This model of dose response, however, remains debated (see, e.g., Kaiser, 2003; Normile, 2011). Yet, under this scenario, at the mechanistic level, beneficial effects of toxins in low concentrations can be the result of compensatory biological processes following an initial disruption in homeostasis (“homeostatic overcompensation,” Calabrese & Baldwin, 2002). At the cellular level, this overcompensation phenomenon includes processes associated with receptor/signaling mechanisms (Calabrese, 2013), DNA damage repair (Schöllnberger, Stewart, Mitchel, & Hofmann, 2004), immune‐function enhancement (Cui et al., 2017), and alteration of gene expression (Sokolov & Neumann, 2015). As epigenetic mechanisms have also been described for hormetic effects (Vaiserman, 2011), a link to environmental matching theory can be made (Kaiser, 2003).

Even if the vast majority of the toxicology literature has not used experimental designs that could be used to test for hormesis (most studies use too few or too high doses as regulatory agencies are most concerned with high‐dose effects, Calabrese & Baldwin, 2000, 2001), hormesis has been found to be present in numerous animal species (Calabrese, 2002; Calabrese & Baldwin, 1998, 2002), and this effect is present for a multitude of carcinogenic substances including environmental pollutants like PAHs and dioxin (Borak & Sirianni, 2006). For example, promising studies have detected hormesis of carcinogenic substances on tumor formation, reproduction, growth, and metabolism (Calabrese & Baldwin, 1998, 2002; Gaya, Akle, Mudan, & Grange, 2015). These effects on cancer development have been extensively discussed in the framework of radiation hormesis, which has been suggested to be one of the mechanisms explaining reduction in cancers at low radiation doses in populations of nuclear bomb survivors (Doss, 2013). At the population level, it is still debated whether hormesis is under natural selection and can evolve in specific types of environments (Costantini, 2014; Parsons, 2001), although genetic variation in hormetic effects and nongenetic inheritance of epigenetic modifications has already been demonstrated (reviewed by Costantini, 2014).

## 5 CONCLUSIONS

We would like to propose that scientists should fully exploit polluted environments as a widespread “natural” field laboratory to detect hormesis responses on cancer development in natural populations. Wild animals are usually exposed to a cocktail of pollutants in low doses, allowing scientists to even test whether and how interactions between these substances might stimulate a hormetic response. Once detected, these beneficial effects might constitute new avenues of research to test whether these substances or combinations of substances can be used to treat cancer.

Because one single method or model cannot thoroughly describe how organisms challenged with EOF resist cancer progression, researchers interested in these forms of responses must engage in greater exchanges and collaborations involving scientists from different disciplines (field and experimental ecologists, eco‐toxicologists, immunologists, evolutionary biologists, oncologists, and pharmacists). As stated above, the interaction networks that link genetic adaptation to phenotypic resistance to cancer are very complex. Nevertheless, we believe that based on the growing available knowledge gained through evolutionary ecotoxicology, and thanks to the rapid advancement of analytical methods, the identification of cancer resistance mechanisms in wildlife through both field and experimental studies is a promising research axis that could bring new insights into cancer treatment. A targeted and systematic phylogenetic approach may be advisable too: Rather than proceeding with scattered and somewhat random case studies across a wide variety of organisms, a structured phylogenetic approach might help guide the search for potentially useful study organisms, for instance, increasingly employed in the field of pharmacologically active plant discovery (e.g., Barbosa et al., 2012). Reciprocally, conservation challenges associated with population decreases due to EOF‐induced carcinogenesis may benefit from human cancer research. It may improve our understanding of the processes involved in contaminant‐induced tumorigenesis, among which some are highly conserved among mammals (Tollis et al., 2017) or even in both mammals and fish (Marchand et al., 2010). At the same time, human cancer research may also be helpful in developing mitigation strategies. These bridges that could lead to fruitful collaborations represent a new step into the One Health approach, which is based on the existing close links between human health, animal health, and ecosystem health and the efforts of biologists, veterinarians, and human health researchers. The One Health approach is increasingly applied in infectious disease studies, but still remains to be incorporated into our understanding of carcinogenesis and the treatment of cancer.

## CONFLICT OF INTEREST

None declared.
